# Early Effects of Metabolic Syndrome on ATP-Sensitive Potassium Channels from Rat Pancreatic Beta Cells

**DOI:** 10.3390/metabo12040365

**Published:** 2022-04-18

**Authors:** Iskra Cruz-Cruz, Germán Bernate-Obando, Carlos Larqué, Rene Escalona, Rodolfo Pinto-Almazán, Myrian Velasco

**Affiliations:** 1 Neuroscience Division, Department of Cognitive Neuroscience, Instituto de Fisiología Celular, Universidad Nacional Autónoma de México, Ciudad Universitaria, AP 70-253 Coyoacán, México City 04510, Mexico; nessuno.23.ic@gmail.com (I.C.-C.); alonsogerman25@gmail.com (G.B.-O.); 2 Embriology and Genetics Deparment, Facultad de Medicina, Universidad Nacional Autónoma de México, Ciudad Universitaria, AP 70-253 Coyoacán, México City 04510, Mexico; skiuty@hotmail.com (C.L.); escalonarj@facmed.unam.mx (R.E.); 3 Non-Communicable Disease Research Group, Facultad Mexicana de Medicina, Universidad La Salle-México, Las Fuentes 17, Tlalpan Centro I, Tlalpan, México City 14000, Mexico; rodolfopintoalmazan@gmail.com

**Keywords:** metabolic syndrome, K_ATP_ channels, glybenclamide, pancreatic beta cell, insulin

## Abstract

Metabolic syndrome (MS) is a cluster of metabolic signs that increases the risk of developing type 2 two diabetes mellitus and cardiovascular diseases. MS leads to pancreatic beta cell exhaustion and decreased insulin secretion through unknown mechanisms in a time-dependent manner. ATP-sensitive potassium channels (K_ATP_ channels), common targets of anti-diabetic drugs, participate in the glucose-stimulated insulin secretion, coupling the metabolic status and electrical activity of pancreatic beta cells. We investigated the early effects of MS on the conductance, ATP and glybenclamide sensitivity of the K_ATP_ channels. We used *Wistar* rats fed with a high-sucrose diet (HSD) for 8 weeks as a MS model. In excised membrane patches, control and HSD channels showed similar unitary conductance and ATP sensitivity pancreatic beta cells in their K_ATP_ channels_._ In contrast, MS produced variability in the sensitivity to glybenclamide of K_ATP_ channels. We observed two subpopulations of pancreatic beta cells, one with similar (Gly1) and one with increased (Gly2) glybenclamide sensitivity compared to the control group. This study shows that the early effects of MS produced by consuming high-sugar beverages can affect the pharmacological properties of K_ATP_ channels to one of the drugs used for diabetes treatment.

## 1. Introduction

In modern society, hyper-caloric diets, sedentary behavior and genetic factors are the most common causes of metabolic syndrome (MS). MS is a constellation of metabolic signs that increases the risk of developing type 2 diabetes mellitus (DM2) and cardiovascular diseases [1]. According to the WHO, MS includes central obesity, dyslipidemias (with reduced high-density lipoprotein cholesterol (HDL-c) and increased triacylglycerols), hypertension, impaired fasting blood glucose, and insulin resistance [2].

Since MS’s definition in 1988, Reaven proposed a strong association of insulin resistance with hyperinsulinemia [3]. Currently, it continues to be debated which of the two occurs first in the pathogenesis of MS. It has been proposed that during MS and DM2, insulin resistance (impaired responses to insulin at target tissues due to alterations in the insulin signaling pathway) increases blood glucose levels. As a consequence, there is an overstimulation of pancreatic beta cells and an increased insulin secretion [4]. Under overnutrition conditions, this compensatory response by beta cells can eventually lead to beta-cell dysfunction and eventually might cause DM2 through mechanisms that are still unknown [5].

Pancreatic beta cells release insulin in response to nutrient intake; however, the glucose-stimulated insulin secretion (GSIS) is the most studied mechanism, which involves multiple metabolic events as well as ion channel activity. Metabolic events start when blood glucose levels increase above basal concentration (>7 mM). In this condition, glucose enters the cell through the glucose transporters (GLUTs), and it is phosphorylated and metabolized (through glycolysis, Krebs cycle, and oxidative phosphorylation), thus increasing the ATP/ADP ratio [6].

The rise in intracellular ATP levels inhibits the ATP-sensitive potassium channel (K_ATP_ channels) activity, reducing the K^+^ efflux, producing a depolarization of the resting membrane potential (approximately −70 mV), increasing the opening probability for voltage-dependent sodium and calcium (T and L types) channels, increasing electrical activity and producing action potentials. The intracellular calcium increase promotes the exocytosis of insulin. Finally, the voltage-dependent potassium channel activity restores the resting membrane potential [6,7].

K_ATP_ channels link the membrane excitability with the metabolic status of pancreatic beta cells. Increased intracellular ATP levels produce a reduction in potassium permeation and enhance membrane excitability. In pancreatic beta cells, functional K_ATP_ channels are hetero-octameric protein complexes composed of four inward rectifier potassium channel 6.2 (Kir6.2) subunits and four sulfonylurea receptors 1 (SUR1) [8]. The Kir6.2 are the pore-forming subunits and act as the ATP sensor, whose activity is regulated by PIP2 [9]. SUR1 subunits regulate the biophysical and pharmacological properties of K_ATP_ channels. In addition, SUR1 subunits are highly sensitive to glybenclamide, one of the most common sulfonylureas used in the DM2 treatment [8,10].

K_ATP_ channels have been poorly studied in the context of MS, a condition where pancreatic beta cells are continuously releasing insulin in an adverse environment with high concentrations of blood glucose and triacylglycerols [11]. Previous works in our laboratory demonstrated that treatment of drinking water with 20% sucrose for 8 and 24 weeks induces metabolic syndrome in rats. Interestingly, the severity of these signs increased with the duration of the HSD treatment [11,12]. We demonstrated that the ATP sensitivity of K_ATP_ channels was higher in the beta cells of rats with long-term MS than that observed in control beta cells [12]. Those changes in K_ATP_ channels could explain the hyperinsulinemia observed during MS. Considering that the MS effects depend on the duration of the HSD treatment, the present study aimed to analyze the early effects of MS on ATP- and glybenclamide-sensitivity of K_ATP_ channels under the hypothesis that the early effects of MS would not affect K_ATP_ channel sensitivity to ATP or glybenclamide.

Interestingly, we observed that short-term MS affects glybenclamide-sensitivity of K_ATP_ channels. This is a highly relevant observation considering the increased prevalence of MS worldwide, which represents a serious public health issue.

## 2. Results

### 2.1. High-Sucrose Diet Induces Obesity

Food and water consumption was quantified by rat per day for both experimental groups. We observed that food consumption was higher in the control group than the high-sucrose diet (HSD) group 23.9 ± 0.3 and 12.6 ± 0.4 g, respectively. In contrast, beverage consumption increased in the HSD group compared to the control group (51.0 ± 1 and 89.2 ± 2 mL, respectively). Table 1 shows the daily caloric intake for the control and HSD groups (*n* = 16 rats for each condition). Total caloric intake was increased in the HSD group (124.7 ± 2 Kcal/day/rat) compared to the control group (96.7 ± 2 Kcal/day/rat).

**Figure 1:**
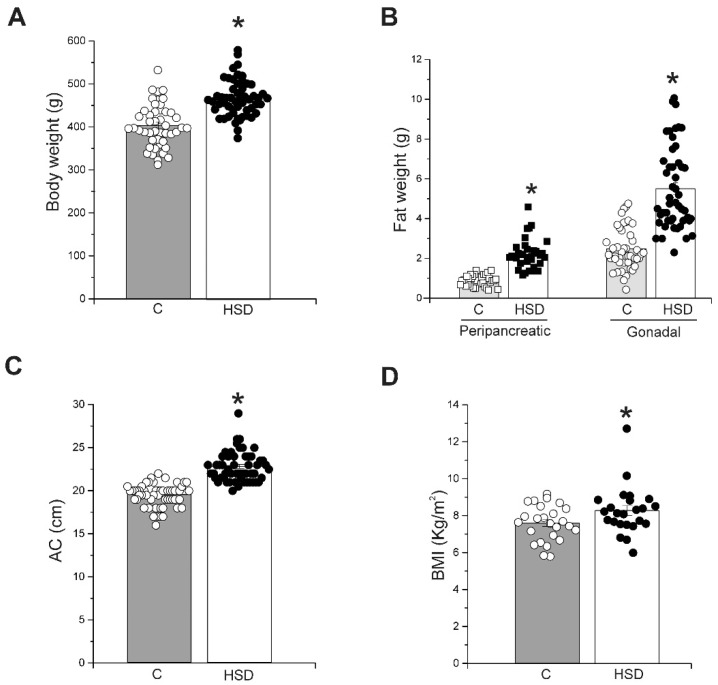
High-sucrose diet induces obesity. (**A**) Body weight gain after 8 weeks on HSD. Bars represent the mean ± SE and each circle or square represents individual data from control (*n* = 46) and HSD rats (*n* = 46). * *p* ≤ 0.0001 compared to the control group. (**B**) Peripancreatic and epididymal fat from control group (grey bars, *n* = 46) and HSD group (white bars, *n* = 46). Bars represent the mean ± SE and each circle or square represents individual data. * *p* ≤ 0.0001 compared to the control group. (**C**) Abdominal circumference (AC) of control and HSD groups. Bars represent the mean ± SE and each circle or square represents individual data. * *p* ≤ 0.0001 compared to control group. (**D**) Body mass index (BMI) of control and HSD groups. Bars represent the mean ± SE and each circle or square represents individual data. * *p* ≤ 0.0001 compared to control group.

The increased caloric intake in the HSD group resulted in an increased body weight gain. After 8 weeks of treatment, the HSD group had a body weight that was 16% higher than that of rats fed with a standard diet (Figure 1A). Moreover, the HSD group increased the weight of the peripancreatic and epididymal white adipose tissue depots (pWAT and eWAT, respectively) by 132% and 129%, respectively, compared to the control group (Figure 1B).

Additionally, the abdominal circumference (AC) and the body mass index (BMI) were analyzed; both obesity-associated parameters were increased in animals with the HSD group. AC increased from 19.4 ± 0.1 cm in the control group to 22.7 ± 0.2 cm in the HSD group (Figure 1C). The HSD group showed a higher BMI compared to the control group (8.5 ± 0.2 and 7.2 ± 0.2 and kg/m^2^, respectively; Figure 1D).

Our data demonstrate that consumption of a high-sucrose diet for 8 weeks induces obesity characterized by an increase in body fat and abdominal circumference, two main criteria for diagnosing MS.

### 2.2. High-Sucrose Diet Changes Metabolic Status

**Figure 2:**
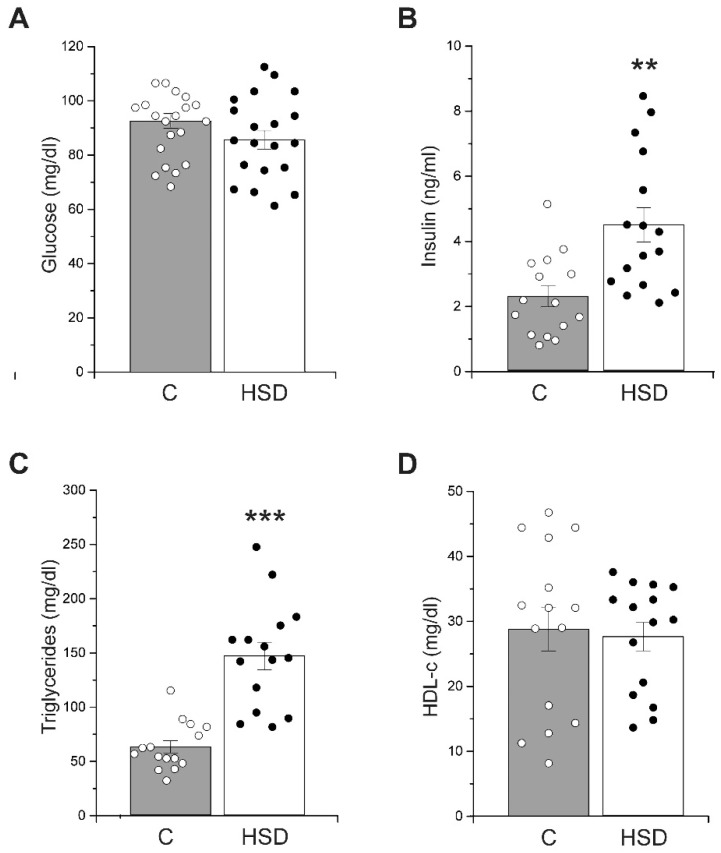
Metabolic status. (**A**) Fasting glucose levels of HSD group compared to the control group. Boxes around the mean represent SE and whiskers represent the SD, *n* = 20 of each condition. (**B**) Plasma insulin levels from control (*n* = 15) and HSD (*n* = 15) groups. Boxes around the mean represent SE and whiskers represent the SD. One-way ANOVA was performed with post hoc Tukey’s test, ** *p* < 0.0001 compared to the control group. (**C**) Plasma triacylglycerols levels from control (*n* = 15) and HSD group (*n* = 15). Boxes around the mean represent the SE, and whiskers represent the SD. One-way ANOVA was performed with post hoc Tukey’s test, *** *p* < 0.01 compared to the control group. (**D**) High-density lipoprotein cholesterol (HDL-c) from control group (*n* = 15) and HSD group (*n* = 15). Boxes around the mean represent SE and whiskers represent the SD.

To assess the effect of a high-sucrose diet on the metabolic status, we quantified fasting plasma glucose, insulin, high-density lipoprotein cholesterol (HDL-c), and triacylglycerols (TG) levels. Plasma glucose levels were similar between control and HSD groups, with 86 ± 3 mg/dL and 81 ± 4 mg/dL, respectively (Figure 2A). Notably, the HSD-fed rats showed an increase in plasma insulin levels (5.06 ± 0.7 mg/L) compared to the control group (2.6 ± 0.5 mg/L) (Figure 2B). Moreover, the TG levels in HSD rats were 131% higher compared to the control group (Figure 2C). In contrast, we did not observe any change in HDL-c levels between control and HSD rats (Figure 2D). Our results show that a high-sucrose diet provokes metabolic alterations related to MS, particularly hyperinsulinemia, which is associated with insulin resistance.

### 2.3. High-Sucrose Diet Produces Insulin Resistance

**Figure 3:**
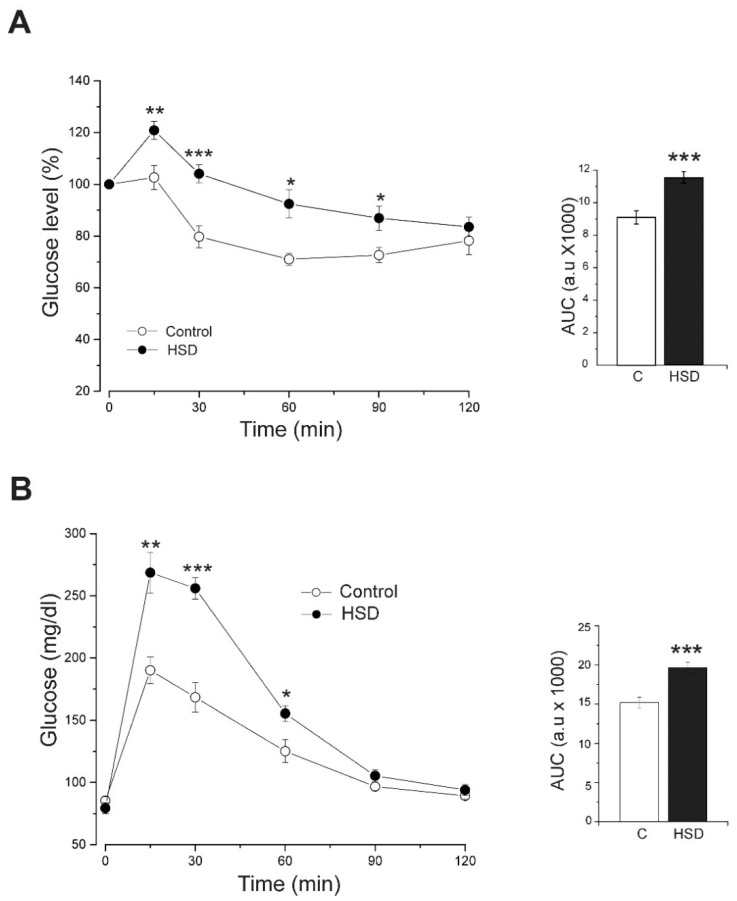
High-sucrose diet induces insulin resistance. (**A**) Left panel, insulin tolerance test performed on fasted overnight control rats (*n* = 13) and HSD rats (*n* = 13). Data expressed as mean ± SE. One-way ANOVA was performed with post hoc Tukey’s test, * *p* < 0.05, ** *p* < 0.01, *** *p* < 0.001 compared to the control group. Right panel, the area under the curve. Bars represent the mean ± SE, *** *p* < 0.001 compared to control group. (**B**) Left panel, intraperitoneal glucose tolerance tests were performed on control (*n* = 21) and HSD (*n* = 14) rats. Data expressed as mean ± SE. One-way ANOVA was performed with post hoc Tukey’s test, * *p* < 0.05, ** *p* < 0.01, *** *p* < 0.0001 compared to the control group. Right panel, the area under the curve. Bars represent the mean ± SE, * *p* < 0.05 compared to the control group.

We performed an insulin tolerance test (ITT) and glucose tolerance test (GTT) in fasted rats for 12 h to assess insulin resistance. After an intraperitoneal dose of insulin (0.2 IU/kg of body weight), we quantified the blood glucose at different time intervals. HSD rats showed higher glucose levels than control rats during almost the entirety of the experiment (15 to 90 min). We also quantified the area under the curve (AUC), which was higher in HSD rats than in control rats (Figure 3A). In the GTT, an intraperitoneal glucose dose (2 g/kg of body weight) produced similar behavior. HSD rats showed a maximum peak at 15 min and higher glucose levels than control rats until 60 min. The AUC from HSD rats was higher than that of control rats (Figure 3B). Both tests showed that HSD induces insulin resistance.

Insulin resistance constitutes a major sign of MS. Our results show that consumption of a high-sucrose diet for 8 weeks induces IR and glucose intolerance in rats. Moreover, our results demonstrate that a high-sucrose diet for 8 weeks induces MS in rats. This animal model could allow further assessment of K_ATP_-channel activity, which is involved in glucose-stimulated insulin secretion.

### 2.4. Early Effects of MS on K_ATP_ Channels Activity

**Figure 4:**
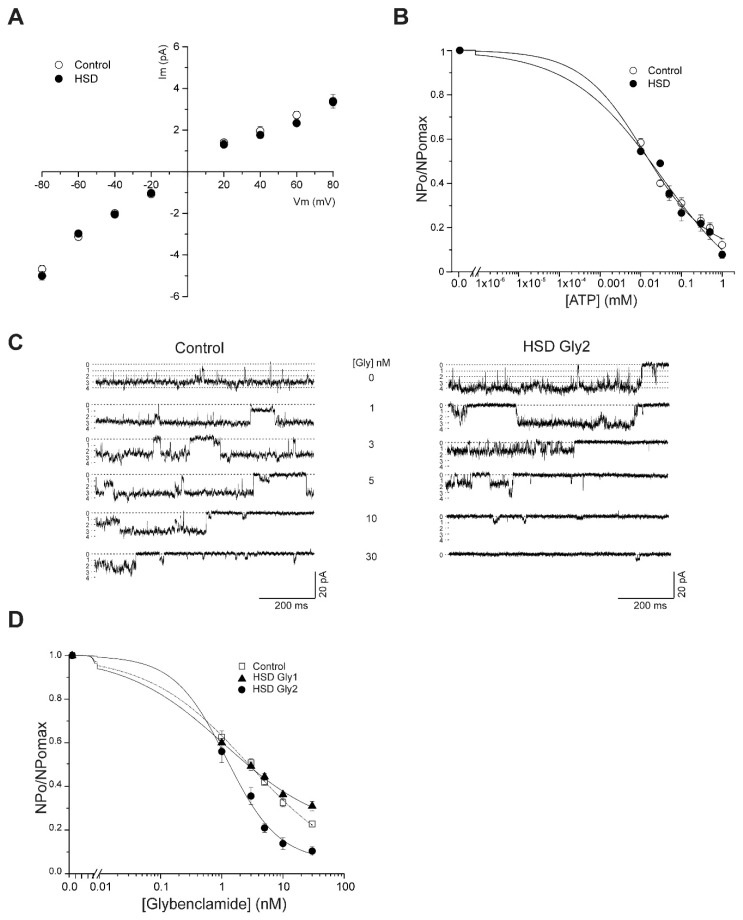
Early effects of MS on ATP and Glybenclamide sensitivity of K_ATP_ channels in beta cells. (**A**) Current to voltage relationship for single channels. Data represent the mean ± SE. The unitary conductance was 56 ± 2 and 59 ± 4 pS for control (*n* = 10) and HSD (*n* = 11), respectively. (**B**) ATP sensitivity of K_ATP_ channels from control and HSD groups. The data were fitted to the Hill equation. IC50 and Hill coefficient were 17.4 ± 0.04 and 0.5 ± 0.04 for control group (*n* = 10), and 18.1 ± 0.001 µM and 0.50 ± 0.07 for the HSD group (*n* = 7), respectively. Data represent the mean ± SE. (**C**) Glybenclamide blocked of K_ATP_ channels activity in a concentration-dependent manner (1, 3, 5, 10 and 30 nM), applied to the cytoplasmic face of an inside-out membrane patches from control and HSD Gly2 (Vm = −60 mV). Different currents levels are indicated to the left of the traces. (**D**) Concentration-response curve to glybenclamide. K_ATP_ channels activity (NPo) normalized (normalized by the maximum) were plotted as a function of glybenclamide concentration and subject to fitting to Hill equation. The IC50 and Hill coefficient (h) for control group (open circle *n* = 10) were 2.8 ± 0.3 nM and 0.5 ± 0.04, respectively. IC50 and h for each subpopulation of the HSD group were 2.8 ± 0.3 nM and 0.4 ± 0.03, respectively, for Gly1 (*n* = 8), and 1.3 ± 0.3 nM and 0.9 ± 0.1, respectively, for Gly2 (*n* = 6). Data are expressed as mean ± SE. One-way ANOVA was performed with post hoc Tukey’s test *p* < 0.05 compared to the control group.

After 8 weeks of treatment with a HSD, rats developed metabolic syndrome. To analyze the early effects of MS on the beta-cell K_ATP_ channel conductance, ATP sensitivity, and glybenclamide sensitivity, we recorded the unitary currents in membrane patches in the inside-out configuration and symmetrical potassium condition. The unitary currents at different membrane potentials were plotted and data fitted to a linear equation (Figure 4A). The unitary conductance was 56 ± 2 and 59 ± 4 pS for control and HSD groups, respectively.

We assessed the ATP-sensitivity of K_ATP_ channels in excised patches from control and HSD pancreatic beta cells at −60 mV membrane potential. We used increasing ATP concentrations (0.01, 0.03, 0.05, 0.1, 0.3, 0.5 and 1 mM), which were added to the high potassium solution, to perfuse the intracellular surface of the K_ATP_ channels. K_ATP_ channel activity (NPo) was reduced with increasing ATP concentrations, and data were adjusted to the Hill equation. We did not observe a difference in the half-maximal inhibitory concentration (IC50) of ATP, 17.4 ± 0.04 and 18.1 ± 0.001 µM to control and HSD groups, respectively. Moreover, the Hill coefficients were 0.5 ± 0.04 and 0.5 ± 0.07 to control and HSD groups, respectively (Figure 4B).

We also analyzed glybenclamide sensitivity in excised patches at −60 mV membrane potential. The application of glybenclamide reduced the K_ATP_ activity in a concentration-dependent manner (Figure 4C). Glybenclamide had distinct effects on the concentration-response curves of control and HSD beta cells. We observed two subpopulations of pancreatic beta cells with different sensitivity to glybenclamide within the HSD group. These were named Gly1 and Gly2. These subpopulations showed an IC50 and Hill coefficient of 2.8 ± 0.3 nM and 0.4 ± 0.03, respectively, for Gly1, and 1.3 ± 0.3 nM and 0.9 ± 0.1, respectively, for Gly2. We found an IC50 and Hill coefficient of 2.8 ± 0.3 nM and 0.5 ± 0.04 for the control group (Figure 4D). These results show that despite development of MS induced by the 8-week high-sucrose diet, K_ATP_-channel activity and their sensitivity to ATP are not affected. It is noteworthy that we identified a beta-cell subpopulation characterized by a decreased sensitivity to glybenclamide.

## 3. Discussion

In this study, we analyzed the early effects of MS on the K_ATP_ channel function. We found that MS induced by a 20% sucrose diet (HSD) for 8 weeks was able to produce changes in the K_ATP_ channels’ affinity to glybenclamide.

Overnutrition and physical inactivity are the major drivers of MS, which is a public health problem worldwide. Its prevalence varies from 10 to 40% [13]. In MS, the crosstalk among central obesity, impaired fasting glucose, hypertension, dyslipidemia, and insulin resistance represents a risk factor for DM2 and cardiovascular diseases. MS is diagnosed by the presence of three of these signs [3,14]. The pathophysiology of MS is complex and includes central obesity, low-grade systemic inflammation, and insulin resistance [14].

We used a metabolic syndrome model in *Wistar* rats, which was previously characterized and is highly reproducible [11]. Our results showed that after 8 weeks of treatment, the HSD group developed central obesity and other features related to MS, such as an increase in BMI, abdominal circumference, and peripancreatic and epididymal fat depots compared to control rats. Furthermore, HSD rats developed hypertriglyceridemia, glucose intolerance, hyperinsulinemia, and insulin resistance.

Insulin resistance has received central attention as the unifying pathophysiological mechanism in MS [15]. Moreover, the pathogenesis of DM2 is best understood as a decrease in insulin secretion due to pancreatic beta-cell exhaustion. In MS, overnutrition and insulin resistance increase glucose levels. This results in hyperinsulinemia due to pancreatic beta-cell hyperfunction with increased insulin biosynthesis and secretion. Consequently, long-term functional overload of pancreatic beta cells and activation of stress-related signals induce progression to beta-cell hypofunction [16].

However, recent studies have observed that some patients show insulin hypersecretion before the development of glucose intolerance, suggesting that hyperinsulinemia can occur before insulin resistance [5,17,18]. Furthermore, hyperinsulinemia contributes to other diseases associated with MS [5]. The dysfunction of pancreatic beta cells has been correlated with the time and severity of MS [19]. Pancreatic beta-cell function during the transition from MS to DM2 is crucial to improve the treatment of both diseases/conditions.

Glucose-stimulated insulin secretion (GSIS) is a complex mechanism through which pancreatic beta cells release insulin in response to increased blood glucose levels. In this mechanism, the glucose metabolism enhances the excitability of pancreatic beta cells through the inhibition of the K_ATP_ channels by ATP, the opening of voltage-dependent sodium and calcium channels, and the increase of intracellular calcium concentration, which triggers insulin secretion [7].

K_ATP_ channels have been widely studied on pancreatic beta cells because mutations in the genes that encode the Kir6.2 (encoded by KCNJ11) and SUR1 (encoded by ABCC8) subunits that form the K_ATP_ channels produce an insulin secretion impairment, leading to the onset of diseases, such as congenital hyperinsulinism [20,21] and neonatal diabetes mellitus [22,23]. In MS, changes in the K_ATP_ channel functions were initially reported by our group [12].

A previous study in our laboratory demonstrated that long-term MS induced changes in the macroscopic calcium currents and the K_ATP_ channel activity. Long-term MS was induced by a longer (24-week) treatment duration with a 20% sucrose diet. The treated rats developed MS with the same signs as those observed in the short-term (8 weeks). However, the 24-week MS model had more severe signs of central obesity, hypertension, hypertriglyceridemia, glucose intolerance, insulin resistance, hyperinsulinemia, and hyperglycemia [1,12]. We did not observe hyperglycemia in the 8-week MS model, indicating that a longer treatment can aggravate the signs of MS.

After 24 weeks of treatment, we found MS beta cells with three calcium current density behavior modes; 50% of the cells had a lower current density, and 35% of the cells had larger current density compared to control cells, and 15% of the cells did not show a calcium current. Furthermore, MS increased the ATP-sensitivity of K_ATP_ channels. We observed that the IC50 to ATP was decreased compared to control cells, indicating that during MS, beta-cell K_ATP_ channels are activated at a lower intracellular ATP concentration than in control cells. This could partially explain the hyperinsulinemia observed after 24 weeks of treatment with HSD [12].

This study analyzed whether short-term MS induces changes in the K_ATP_ channel function or not. After 8 weeks of treatment, we did not observe changes in the ATP sensitivity of K_ATP_ channels compared to control cells. However, when we analyzed their sensitivity to glybenclamide, we identified two subpopulations of pancreatic beta cells with similar (Gly1) and higher (Gly2) glybenclamide sensitivity compared to control cells. These results are interesting because when we compared the short-term to long-term MS effects, we observed that the longer exposure time to an HSD resulted in impaired K_ATP_ channel function. Moreover, our results also showed that short-term MS induced changes in the sensitivity of glybenclamide of the K_ATP_ channels.

A limitation of the present work is the lack of further exploration of molecular mechanisms that impair K_ATP_ channel function in MS. Metabolic syndrome is complex and involves several metabolic signs, which include impairment of lipid metabolism. Dyslipidemias could play an important role in these effects observed on K_ATP_ channels. There is evidence that suggests that lipids such as phosphatidylinositol-4, 5-biphosphate (PIP2), long-chain acyl-CoA esters (LC-CoAs), and palmitoyl CoA can modulate the K_ATP_-channel ATP- [24,25] and glybenclamide-sensitivity [24,26].

The effects of PIP2 and phosphatidylinositol-3, 4, 5-triphosphate over excised oocyte membrane patches reduced the K_ATP_ channel sensitivity to ATP and glybenclamide. Interestingly, this effect was reversed when the patch was inserted back into the oocyte, suggesting a reversible modulation through endogenous PIPs-degrading enzymes [24]. Furthermore, in excised membrane patches, K_ATP_ channels showed variability in the sensitivity to glybenclamide [24]. Moreover, acyl-CoA esters, lipids, and oleates interact with the sulphonylurea receptor, modulating the sulphonylurea binding rate [26]. This evidence is of pathological relevance since obesity and long-term high-fat diets induce changes in membrane-lipids composition [27]. In addition, it is possible that MS could induce an unknown phosphorylation that could modify the glybenclamide binding site at the SUR1 of the K_ATP_ channel, which could result in a higher sensitivity to glybenclamide such as that observed in the Gly2 beta-cell subpopulation. Moreover, we identified two subpopulations of beta cells with different glybenclamide sensitivity. However, whether they have any other morphological or physiological differences remains to be determined. Finally, the mechanism involved in this differential susceptibility to the effects of MS over pancreatic beta cells remains to be determined.

In conclusion, our data contribute to the idea that overnutrition and excess of carbohydrates, in addition to the adverse environment of MS, can overstimulate pancreatic beta cells and eventually lead to the exhaustion and failure of pancreatic beta cells. Moreover, this work shows that short-term MS may affect the glybenclamide binding to K_ATP_ channels and impair the beta-cell response to DM2 treatment. The presence of two cell populations with different sensitivity to glybenclamide suggests that not all beta cells are equally susceptible to MS. The elucidation of the mechanisms for this heterogenicity requires further research.

## 4. Materials and Methods

Reagents were obtained from the following sources: Hank’s balanced salt solution, RPMI-1640 medium, fetal bovine serum (FBS), and penicillin-streptomycin-amphotericin B solution were purchased form Thermo Fisher Scientific (Waltham, MA, USA). 4-(2-hydroxyethyl)-1-piperazineethanesulfonic acid (HEPES), poly-L-lysine, ethylene glycol-bis [β-aminoethylether] *N*,*N*,*N′*,*N′*-tetraacetic acid] (EGTA), and collagenase from *Clostridium histolyticum*, glybenclamide, adenosine 5′-triphosphate potassium salt were purchased from Merck (Darmstadt, Germany); bovine serum albumin (BSA) and gentamicin reagent were purchased from Microlab Laboratory (CDMX, México).

### 4.1. Animals

All methods used in this study were approved by the Animal Care Committee of the Instituto de Fisiología Celular (protocol number MHU-69-15), Universidad Nacional Autónoma de México. Animal care was performed according to the “International Guiding Principles for Biomedical Research Involving Animals”, Council for International Organizations of Medical Sciences, 2010.

### 4.2. Metabolic Syndrome Model

We used young adult (250 to 280 g, approximately 8 weeks of age) male *Wistar* rats, obtained from the local facility of the Instituto de Fisiología Celular Universidad Nacional Autónoma de México. Rats were randomly assigned to control and treated groups and were housed in a 12 h light/dark cycle, at 20–23 °C with 40% relative humidity. All animals were fed with standard rat chow (Lab Diet 5001) with a caloric composition of 28.5% protein, 13.5% fat, and 58% carbohydrates (4.07 Kcal/gram). *Ad libitum* tap water was provided to the control group, or 20% (*w*/*v*) sucrose solution (high-sucrose diet, HSD) to the treated group, for 8 weeks as previously described [1,11].

All measurements and experiments were performed on rats that fasted for 12 h, a period during which the sucrose solution was replaced with tap water. At the end of the treatment, animals were anesthetized with an intraperitoneal (IP) injection of sodium pentobarbital (40 mg/kg).

We obtained blood samples from the inferior cava vein in heparinized tubes and centrifuged to obtain plasma, which was stored at −20 °C for posterior use. Additionally, pancreatic tissue, peripancreatic and gonadal fat depots were dissected. Animals were euthanized with a pentobarbital (100–150 mg/kg) overdose.

### 4.3. Insulin Tolerance Test (ITT) and Intraperitoneal Glucose Tolerance Test (IPGTT)

We performed the ITT or IPGTT on animals that fasted for 12 h, one week before the end of the treatment. An intraperitoneal (ip) insulin (0.2 UI/kg of body weight) or glucose (2 g glucose/kg of body weight) injection was administered to each rat. In order to minimize stress, the animals were handled by the same person. Peripheral blood samples were collected from the tail vein immediately before glucose or insulin administration (time 0) and 15, 30, 60, and 120 min after the injection. Glucose levels were determined with a glucose reagent strip and a glucometer (Accu-Check, Hoffman La Roche, Basel, Switzerland). During the ITT, animals that showed glucose levels under 40 mg/dL were fed immediately and excluded from the test.

### 4.4. Somatometric Parameters

After 8 weeks, we determined the following measures: body weight (g), abdominal circumference (AC), and body length (BL). AC was obtained by measuring the abdominal region’s diameter, just above the iliac crest, whereas BL was obtained by measuring the animal length from the tip of the nose to the anus.

### 4.5. Islet Cell Culture

Islet cells were obtained as described previously [12,24,28]. Briefly, pancreatic islets were isolated by collagenase digestion and handpicked from the acinar tissue. The dissociation of the cells was achieved by incubating them in a shaking bath for 5 min at 37 °C in calcium-free Spinner solution, with 10 mM glucose, 0.5% bovine serum albumin, and 3 mM EGTA (ethylene glycol-bis (β-aminoethylether) *N*,*N*,*N′*,*N′*—tetraacetic acid), followed by mechanical disruption. Single cells were cultivated in RPMI-1640 (11.6 mM glucose) supplemented with 0.1% glutamine, 10% fetal bovine serum, 200 units/mL penicillin G, 200 μg/mL streptomycin, and 0.5 μg/mL amphotericin B.

### 4.6. Metabolic Parameters

Glucose levels were determined with a glucose reagent strip and a glucometer (Accu-Check, Hoffman La Roche, Basel, Switzerland) in peripheral blood samples from the tail vein. Plasma insulin levels were determined using the Ultrasensitive rat insulin ELISA system according to manufacturer’s instructions (10-1137-10; Mercodia, Uppsala, Sweden). Triacylglycerols were determined in plasma by colorimetric glycerol phosphate oxidase and phenol 4-amine anti-pyrene peroxidase (GOP-PAP) method (RANDOX laboratories), using a Random RX Imola, according to the manufacturer’s instruction (TR 3823, Randox Laboratories Limited, Crumlin, UK). High-density lipoprotein cholesterol (HDLc) was determined in plasma using a colorimetric method according to the manufacturer’s instruction.

### 4.7. Electrophysiology

All electrophysiological measurements were performed at room temperature (20–22 °C) on pancreatic beta cells from control and HSD groups. Inside-out configuration of Patch Clamp techniques [29] was used to record the K_ATP_ single-channel currents. Data were acquired using an Axopatch 200 A amplifier and Digidata 1322 A digitizer with pClamp 9.0 software (Molecular Devices) and stored for subsequent analysis.

Pipettes were pulled from capillary tubes KIMAX-51 (Kimble Glass Vineland, NJ, USA) to a resistance of 6–10 MΩ when filled with a high-potassium solution containing (in mM): 140 KCl, 1 MgCl_2_, 5 EGTA, and 10 HEPES (pH 7.2 with KOH). Pipette tips were coated with dental wax. Twenty-four hours after seeding, cells were placed in a recording chamber with the standard solution (in mM): 135 NaCl, 5 KCl, 1 MgCl_2_, 10 HEPES, and 10 glucoses (pH adjusted to 7.4 with NaOH). After gigaseal achievement, patches were excised, and the internal membrane face was bathed with the high-potassium solution.

K_ATP_ single currents were digitized at 10 kHz and low-pass filtered at 2 kHz. For the current–voltage relationship, we used current records of a 30 sec duration. The concentrations–response relationships were determined at membrane potential of −60 mV, using adenine 5′-triphosphate potassium salts and glybenclamide added to the high-potassium solution at the indicated concentrations, fitting the experimental values to the Hill equation: Po/Pomax = (1/1 + (x/IC50)^h^), where Po is the open probability of the K_ATP_ channels, Pomax is the maximum open probability, x is the concentration of ATP or glybenclamide, IC50 is the half-blocking concentration, and h is the Hill coefficient or slope factor defined as the steepness of the curve.

### 4.8. Statistical Analysis

All data are reported as the mean ± SE; *n* denotes the number of animals or cells analyzed. The statistical significance for *p* value < 0.05 was obtained with one-way analysis of variance (ANOVA) with post hoc Tukey’s test (Origin 2016 software).

## Figures and Tables

**Table 1:** Daily caloric intake and nutrimental distribution.

Nutrients	Control Kcal/Day/Rat (% of Daily Intake)	MS Kcal/Day/Rat (% of Daily Intake)
Carbohydrates	54.9 (56%)	103 (83%)
Proteins	27.2 (29%)	14.8 (12%)
Fat	13.05 (13%)	6.9 (5%)
Total	96.7 ± 1 (99%)	124.7 ± 2 (99%) *

Data are expressed as mean ± SEM. * *p* ≤ 0.001 compared to control group.

## Data Availability

The data presented in this study are available in article.

## References

[1] Velasco M., Ortiz-Huidobro R.I., Larque C., Sanchez-Zamora Y.I., Romo-Yanez J., Hiriart M. (2020). Sexual Dimorphism in Insulin Resistance in a Metabolic Syndrome Rat Model. Endocr. Connect..

[2] Grundy S.M., Neeland I.J., Turer A.T., Vega G.L. (2014). Ethnic and Gender Susceptibility to Metabolic Risk. Metab. Syndr. Relat. Disord..

[3] Reaven G.M. (1988). Banting Lecture 1988. Role of Insulin Resistance in Human Disease. Diabetes.

[4] Prentki M., Nolan C.J. (2006). Islet Beta Cell Failure in Type 2 Diabetes. J. Clin. Investig..

[5] Nolan C.J., Prentki M. (2019). Insulin Resistance and Insulin Hypersecretion in the Metabolic Syndrome and Type 2 Diabetes: Time for a Conceptual Framework Shift. Diabetes Vasc. Dis. Res..

[6] Velasco M., Diaz-Garcia C.M., Larque C., Hiriart M. (2016). Modulation of Ionic Channels and Insulin Secretion by Drugs and Hormones in Pancreatic Beta Cells. Mol. Pharmacol..

[7] Hiriart M., Aguilar-Bryan L. (2008). Channel Regulation of Glucose Sensing in the Pancreatic Beta-Cell. Am. J. Physiol. Endocrinol. Metab..

[8] Aguilar-Bryan L., Bryan J. (1999). Molecular Biology of Adenosine Triphosphate-Sensitive Potassium Channels. Endocr. Rev..

[9] Baukrowitz T., Schulte U., Oliver D., Herlitze S., Krauter T., Tucker S.J., Ruppersberg J.P., Fakler B. (1998). Pip2 and Pip as Determinants for Atp Inhibition of Katp Channels. Science.

[10] Tucker S.J., Gribble F.M., Zhao C., Trapp S., Ashcroft F.M. (1997). Truncation of Kir6.2 Produces Atp-Sensitive K+ Channels in the Absence of the Sulphonylurea Receptor. Nature.

[11] Larque C., Velasco M., Navarro-Tableros V., Duhne M., Aguirre J., Gutierrez-Reyes G., Moreno J., Robles-Diaz G., Hong E., Hiriart M. (2011). Early Endocrine and Molecular Changes in Metabolic Syndrome Models. IUBMB Life.

[12] Velasco M., Larque C., Gutierrez-Reyes G., Arredondo R., Sanchez-Soto C., Hiriart M. (2012). Metabolic Syndrome Induces Changes in Katp-Channels and Calcium Currents in Pancreatic Beta-Cells. Islets.

[13] Grundy S.M. (2016). Metabolic Syndrome Update. Trends Cardiovasc. Med..

[14] Rochlani Y., Pothineni N.V., Kovelamudi S., Mehta J.L. (2017). Metabolic Syndrome: Pathophysiology, Management, and Modulation by Natural Compounds. Ther. Adv. Cardiovasc. Dis..

[15] Balkau B., Charles M.A. (1999). Comment on the Provisional Report from the Who Consultation. European Group for the Study of Insulin Resistance (Egir). Diabet. Med..

[16] Cerf M.E. (2020). Beta Cell Physiological Dynamics and Dysfunctional Transitions in Response to Islet Inflammation in Obesity and Diabetes. Metabolites.

[17] Corkey B.E. (2012). Diabetes: Have We Got It All Wrong? Insulin Hypersecretion and Food Additives: Cause of Obesity and Diabetes?. Diabetes Care.

[18] (2012). Banting Lecture 2011: Hyperinsulinemia: Cause or Consequence?. Diabetes.

[19] Hudish L.I., Reusch J.E., Sussel L. (2019). Beta Cell Dysfunction During Progression of Metabolic Syndrome to Type 2 Diabetes. J. Clin. Investig..

[20] Dunne M.J. (2000). Ions, Genes and Insulin Release: From Basic Science to Clinical Disease. Based on the 1998 R. D. Lawrence Lecture. Diabet. Med..

[21] Shah P., Demirbilek H., Hussain K. (2014). Persistent Hyperinsulinaemic Hypoglycaemia in Infancy. Semin. Pediatr. Surg..

[22] Edghill E.L., Flanagan S.E., Ellard S. (2010). Permanent Neonatal Diabetes Due to Activating Mutations in Abcc8 and Kcnj11. Rev. Endocr. Metab. Disord..

[23] Remedi M.S., Koster J.C. (2010). K(Atp) Channelopathies in the Pancreas. Pflug. Arch..

[24] Krauter T., Ruppersberg J.P., Baukrowitz T. (2001). Phospholipids as Modulators of K(Atp) Channels: Distinct Mechanisms for Control of Sensitivity to Sulphonylureas, K(+) Channel Openers, and Atp. Mol. Pharmacol..

[25] Schulze D., Rapedius M., Krauter T., Baukrowitz T. (2003). Long-Chain Acyl-Coa Esters and Phosphatidylinositol Phosphates Modulate Atp Inhibition of Katp Channels by the Same Mechanism. J. Physiol..

[26] Klein A., Lichtenberg J., Stephan D., Quast U. (2005). Lipids Modulate Ligand Binding to Sulphonylurea Receptors. Br. J. Pharmacol..

[27] Wilcox G. (2005). Insulin and Insulin Resistance. Clin. Biochem. Rev..

[28] Velasco M., Larque C., Diaz-Garcia C.M., Sanchez-Soto C., Hiriart M. (2018). Rat Pancreatic Beta-Cell Culture. Methods Mol. Biol..

[29] Hamill O.P., Marty A., Neher E., Sakmann B., Sigworth F.J. (1981). Improved Patch-Clamp Techniques for High-Resolution Current Recording from Cells and Cell-Free Membrane Patches. Pflügers Arch..

